# Recovery of an injured arcuate fasciculus via transcallosal fiber in a stroke patient: A case report

**DOI:** 10.1097/MD.0000000000026840

**Published:** 2021-08-06

**Authors:** Sung Ho Jang, Jeong Pyo Seo, Young Hyeon Kwon

**Affiliations:** a Department of Physical Medicine and Rehabilitation, College of Medicine, Yeungnam University 317-1, Daemyungdong, Namku, Daegu, Republic of Korea; b Department of Physical Therapy, College of Health Sciences, Dankook University Cheonan, Korea, Republic of Korea; c Department of Physical Medicine and Rehabilitation, College of Medicine, Yeungnam University 317-1, Daemyungdong, Namku, Taegu, Republic of Korea.

**Keywords:** aphasia, arcuate fasciculus, diffusion tensor imaging, diffusion tensor tractography, stroke

## Abstract

**Rationale:**

We report on a patient whose arcuate fasciculus (AF) and corticobulbar tract (CBT) recovered following an infarct in the middle cerebral artery (MCA) territory, demonstrated on serial diffusion tensor tractography (DTT).

**Patient concerns:**

The patient showed moderate conduction aphasia on the Western Aphasia Battery with an aphasia quotient of 46.5‰ (spontaneous speech: 35.0‰, auditory comprehension: 36.0‰, and naming: 53.1‰) at 1 month after onset. His aphasia improved with an aphasia quotient of 49‰ (spontaneous speech: 71.0‰, auditory comprehension: 52.0‰, and naming: 59.0‰) at 10 months after onset.

**Diagnosis:**

A 44-year-old right-handed male patient presented with aphasia and quadriplegia, which occurred at the onset of an infarct in the left MCA territory.

**Intervention:**

Diffusion tensor imaging data were acquired twice (1 month and 10 months after onset).

**Outcomes:**

On one-month DTT, the discontinuation of the left AF and severe narrowing of the right CBT were observed. However, on ten-month DTT, the left AF was connected to the opposite AF by a new tract that passed through the splenium of corpus callosum, and the right CBT had become thicker.

**Lessons:**

We believe that our results suggest a recovery mechanism of injured AF and CBT in stroke patients.

## 1 Introduction

The arcuate fasciculus (AF) is a neural tract connecting Broca and Wernicke areas, and the corticobulbar tract (CBT) innervates muscles of the face, tongue, jaw, and pharynx, via the cranial nerves.^[1,2]^ These neural tracts are involved in language function; therefore injury of these neural tracts causes a number of language problems.^[3,4]^ However, little is known about the mechanism for recovery of language deficit.^[5–8]^

Aphasia, an acquired neurogenic communication disorder defined by deficits in language comprehension and production, is one of the most common and devastating sequelae of stroke.^[9]^ Approximately 20% to 30% of stroke patients have suffered from aphasia, and more than 10% of stroke patients suffer from chronic aphasia.^[10,11]^ Therefore, elucidation of the recovery mechanism of aphasia is important in stroke patients.

Diffusion tensor tractography (DTT), derived from diffusion tensor imaging (DTI), enables three-dimensional visualization and estimation of the AF and CBT.^[9,12,13]^ Many DTT studies report on injury of the AF and CBT in various brain pathologies.^[9,12–14]^ However, only a few DTT studies report on recovery of an injured AF in patients with brain injury, and no study reported recovery of an injured CBT.^[5,8,15]^

In the current study, we report on a patient whose injured AF and CBT recovered following an infarct in the middle cerebral artery (MCA) territory, demonstrated on serial DTTs.

## 2 Case report

**Figure 1:**
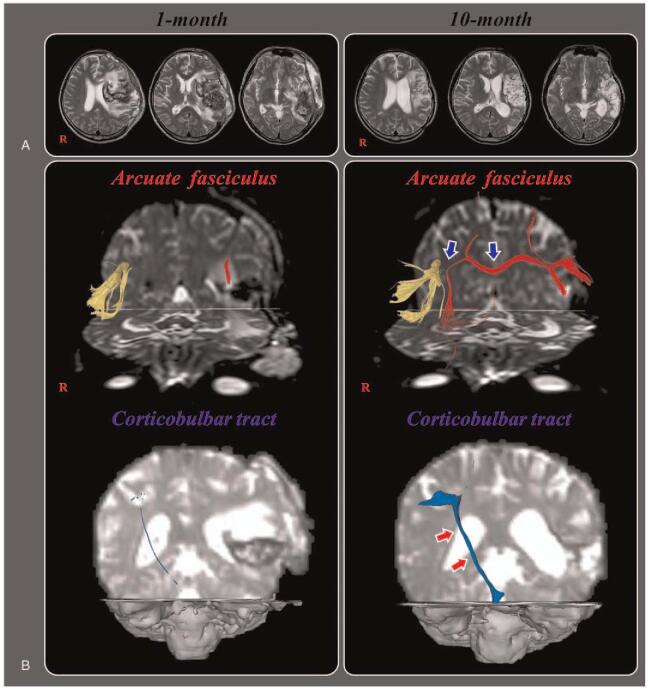
(A) Brain MR images at 1 month after onset show an infarct in the left middle cerebral artery territory, hemorrhagic transformation and subfalcine herniation. Brain MR images at 10 months after onset reveal leukomalactic lesions in the left fronto-parieto-temporo-occipital areas. (B) Results of diffusion tensor tractography (DTT) for the arcuate fasciculus (AF) and corticobulbar tract (CBT). On one-month DTT, the discontinuation of the left AF and severe narrowing of the right CBT are observed. By contrast, on 10-month DTT, the left AF is connected to opposite AF by a new tract that passed through the splenium of corpus callosum (blue arrows) and the right CBT become thicker (red arrows).

A 44-year-old right-handed male patient presented with aphasia, poor awareness and quadriplegia, which occurred at the onset of an infarct in the left MCA territory. He underwent decompressive craniectomy and extra-ventricular drainage catheterization (EVD) due to hemorrhagic transformation and progression of brain swelling at the department of neurosurgery of a university hospital. A brain MRI taken 1 month after EVD showed leukomalactic lesions in the left fronto-parieto-temporo-occipital areas (Fig. 1-A). The Western Aphasia Battery was used to evaluate the patient's language dysfunction.^[16]^ The patient showed moderate conduction aphasia on the K-WAB with an aphasia quotient of 46.5‰ (spontaneous speech: 35.0‰, auditory comprehension: 36.0‰, and naming: 53.1‰) at 1 month after onset. He was transferred to the department of rehabilitation and underwent rehabilitative therapy including speech therapy for 2 months. He was discharged to a local rehabilitation hospital and received similar rehabilitative management until 10 months after the EVD. His aphasia improved, with an aphasia quotient of 49‰ (spontaneous speech: 71.0‰, auditory comprehension: 52.0‰, and naming: 59.0‰). The patient provided signed, informed consent, and the study protocol was approved by the institutional review board of our university hospital (ethical approval number: YUMC-2021-03-014).

## 3 Diffusion tensor imaging

DTIs were obtained twice (1 month and 10 months after onset). DTI was performed using a sensitivity-encoding head coil on a 1.5-T Philips Gyroscan Intera (Hoffman-LaRoche Ltd, Best, The Netherlands) with single-shot echo-planar imaging and navigator echo. Sixty-5 contiguous slices (acquisition matrix = 96 × 96; reconstruction matrix = 192 × 192; field of view = 240 × 240 mm^2^; TR = 10,726 ms; TE = 76 ms, b = 1,000 s/mm^2^, NEX = 1, and thickness = 2.5 mm) were acquired for each of the 32 non-collinear diffusion-sensitizing gradients. For reconstruction of the AF, fiber tracking was performed using the fiber assignment continuous tracking (FACT) algorithm implemented within the DTI task card software (Philips Extended MR Work Space 2.6.3). Each of the DTI replications was intra-registered to the baseline “b0” images to correct for residual eddy-current image distortions and head motion effect, using a diffusion registration package (Philips Medical Systems). The seed region of interest (ROI) was placed manually in the deep white matter of the posterior parietal portion of the superior longitudinal fascicle and the target ROI was placed on the posterior temporal lobe.^[8]^ Fiber tracts passing through both ROIs were designated as the final tracts of interest. The termination criteria used for fiber tracking were FA < 0.15, angle < 27^o^.^[8]^ The CBT was analyzed with the Oxford Centre for Functional Magnetic Resonance Imaging of the Brain Software Library (FSL; www.fmrib.ox.ac.uk/fsl), Affine multi-scale two-dimensional registration corrected for head motion effect and image distortion due to eddy current. A probabilistic tractography method, based on a multifiber model, was used for fiber tracking, which was applied utilizing tractography routines implemented in functional magnetic resonance imaging of the brain diffusion (5000 streamline samples, 0.5 mm step lengths, curvature thresholds = 0.2).^[17]^ The seed ROI was placed on the portion of the CBT area (between transverse pontine fibers and the middle cerebellar peduncle) at the level of mid pons with the axial slice. The target ROI was placed on the lower portion of the precentral gyrus and in the section of the top of the lateral ventricles.^[12]^

On one-month DTT, discontinuation of the left AF and severe narrowing of the right CBT were observed. However, on ten-month DTT, the left AF was connected to opposite AF by a new tract that passed through the splenium of corpus callosum and the right CBT became thicker (Fig. 1-B). The left CBT was not reconstructed on either 1 or ten-month DTTs (Fig. 1-B).

## 4 Discussion

In the current study, we observed the recovery of AF, apparently injured by an MCA infarct, and CBT, apparently injured by a subfalcine herniation, with improvement of language function during a 9 month period in a patient with cerebral infarct. The discontinued left AF was connected to the opposite AF by a new tract that passed through to the splenium of the corpus callosum, and the injured right CBT became thicker. Thickening of the right CBT appeared to indicate recovery of the injured CBT. The patient's language function improved, especially spontaneous speech (1 month: 35.0‰ - > 10 month: 71.0‰). We hypothesize that the improvement of spontaneous speech is related to the new language pathway following injures of the AF and CBT in the left hemisphere: the left AF- > transcallosal fibers - > right AF - > right CBT.

After development of DTI, a few studies reported on recovery mechanism of the AF.^[5,8]^ In 2009, Schlaug et al found increases of the fiber number and volume of the right AF with improvement of speech ability in 6 chronic stroke patients with Broca aphasia.^[5]^ Jang et al [2014] recently reported that the discontinued left AF was elongated to the left Broca area with improvement of aphasia in a patient with an intracerebral hemorrhage.^[8]^ To the best of our knowledge, this is the first study to demonstrate recovery of the AF via a transcallosal fiber and the CBT concurrent improvement of language function. However, a limitation of DTT is that it may underestimate the fiber tracts because regions of fiber complexity and crossing can prevent full reflection of the underlying fiber architecture by DTI. Thus, readers should exercise caution in interpretation.

In conclusion, recovery of an injured AF via transcallosal fibers and injured CBT along with improvement of aphasia was demonstrated in a patient with cerebral infarct. We believe that our results suggest a recovery mechanism of injured AF and CBT in stroke patients. Further complementary studies involving larger numbers of patients are warranted.

## Author contributions

**Conceptualization:** Sung Ho Jang, Jeong Pyo Seo.

**Data curation:** Jeong Pyo Seo, Younghyeon Kwon.

**Formal analysis:** Jeong Pyo Seo.

**Investigation:** Jeong Pyo Seo, Younghyeon Kwon.

**Methodology:** Jeong Pyo Seo, Younghyeon Kwon.

**Software:** Younghyeon Kwon.

**Supervision:** Sung Ho Jang.

**Writing – original draft:** Sung Ho Jang, Jeong Pyo Seo, Younghyeon Kwon.

**Writing – review & editing:** Sung Ho Jang, Jeong Pyo Seo, Younghyeon Kwon.
